# An In Vitro Analysis of Implant Site Preparation and Placement Protocols on Implant Accuracy in Robot-Assisted Procedures

**DOI:** 10.3390/dj13120592

**Published:** 2025-12-10

**Authors:** Yunxiao Wang, Yulan Wang, Richard J. Miron, Yufeng Zhang, Qi Yan

**Affiliations:** 1 State Key Laboratory of Oral & Maxillofacial Reconstruction and Regeneration, Key Laboratory of Oral Biomedicine Ministry of Education, Hubei Key Laboratory of Stomatology, School & Hospital of Stomatology, Wuhan University, Wuhan 430079, China; yunxiaowang@whu.edu.cn (Y.W.); wyl.dentistry@whu.edu.cn (Y.W.); 2 Department of Oral Implantology, School & Hospital of Stomatology, Wuhan University, Wuhan 430079, China; 3 Department of Periodontology, University of Bern, 3012 Bern, Switzerland; 4 Oral Biomaterials and Application Technology Engineering Research Center of Hubei Province, Wuhan 430079, China

**Keywords:** computer-assisted surgeries, dental implantation, guided surgery, accuracy, mechanical stress

## Abstract

**Background/Objectives**: To determine the optimal site preparation and placement protocols for immediate implant positioning in robot-assisted surgeries. **Methods**: In vitro models of immediate and healed extraction sockets were created using 3D printing. A robotic system was used for implant site preparation and implant placement. The implant surgeries were allocated into eight experimental groups using 12 printed models in total. Each model incorporated two implant sites, an immediate site (tooth 21) and a healed site (tooth 26), resulting in 24 implants overall. With 3 implants assigned to each group, the 24 implant placements were evenly distributed across the 8 groups. For each group, the lateral force experienced during surgery was recorded by the haptic sensor on the robotic arm, and implant positional deviations were assessed by superimposing post-surgical CBCT images with the virtual implant planning. **Results**: Healed sites showed significantly higher accuracy than immediate sites, with reduced platform and apical deviations (*p* < 0.001) and markedly lower lateral force experienced by drills. In fully guided procedures, thread tapping greatly improved accuracy in immediate sites but had limited benefit in healed sites. Compared with partially guided workflows, fully guided rCAIS markedly enhanced accuracy in immediate sites (≈0.8 mm reduction in platform/apical deviation, *p* < 0.001), while no meaningful differences were observed in healed sites. Fully guided protocols also reduced insertion force in healed sites. **Conclusions**: Immediate sites showed lower implant positional accuracy and experienced higher lateral forces during surgery than healed sites. In immediate sites, thread tapping and fully guided rCAIS improved placement accuracy.

## 1. Introduction

Improper three-dimensional implant positioning can lead to a variety of problems, especially when implants are placed more buccally since the buccal bone plate is thinner/less dense, which may then lead to buccal bone resorption and gingival recession, an aesthetically displeasing result [1,2,3]. The accuracy of implant placement has a significant impact on the long-term survival and aesthetic outcome of the implants. Implant positional accuracy is assessed using three main metrics: angular deviation, global platform deviation, and global apical deviation. These metrics are measured by comparing the planned implant position to the actual position using 3D imaging, typically CBCT.

Recently, computer-aided implant surgery (CAIS) has significantly enhanced the accuracy of dental implant placement, yet certain factors such as bone density variations or bone crest width can still provoke errors, particularly in immediate implant placement scenarios. The utilization of static surgical guides or dynamic navigation systems has generally improved the accuracy of implant positioning, yet to this day these methods do not completely eliminate deviations. Studies indicate that variables such as the inhomogeneity of bone density or bone slope, particularly in immediate versus healed socket implant placement, can affect the accuracy of these digital methods. A study conducted by Koticha et al. found that implants placed immediately consistently exhibited a tendency to deviate buccally from their intended insertion direction [3].

Robot-assisted dental implant surgery combines advanced imaging techniques with computer-aided design (CAD) software for precise preoperative planning [4]. It utilizes robotic arms to execute accurate drilling and implant placement, supported by real-time navigation systems to ensure implants are placed precisely according to the predetermined plan [5], thereby reducing/minimizing human error [6]. Although this technology is still evolving, it has shown potential to enhance the accuracy and efficiency of implant surgeries in clinical trials investigating its application [7,8,9]. Moreover, the force feedback capabilities of robotic arms were shown to help quantify/reduce lateral forces during implant surgery, especially during immediate implant placement [8]. Furthermore, it has been hypothesized that the utilization of robot-assisted dental implant placement will reduce the effects of lateral force on buccal malposition of implants during implant placement, potentially favoring long-term implant success.

One major reported difference during implant placement procedures among different implant systems is whether thread tapping is required. Implant tapping refers to the final step in the preparation of the implant socket, where a tapping drill is used to prepare and match threads on the implant surface with those in the socket. However, there is currently no reported evidence regarding the influence of different drilling sequences/protocols on the accuracy of implant position during immediate implant placement.

Fully guided rCAIS (FG) implant procedures have been considered more precise in both static and dynamic navigation surgeries, thereby providing more precise control throughout the entire process compared to freehanded or partially guided procedures. FG surgeries have demonstrated superior accuracy in implant positioning, particularly at the apex of the implant [10]. During implant site preparation and implant insertion, lateral forces may influence the final three-dimensional position of the implant. Previous studies have suggested that uncontrolled lateral forces during drilling or insertion may contribute to positional deviations [3]. While robotic systems ensure high reproducibility in planned trajectories, their ability to modulate insertion forces in real time remains under ongoing investigation. In contrast, partially guided workflows allow clinicians to manually control insertion forces, which may theoretically influence accuracy under certain conditions. However, whether manual or robotic insertion offers superior precision remains unclear and requires experimental evaluation.

Given these knowledge gaps, the present in vitro study used a robotic dental implant system to investigate how bone morphology (immediate vs. healed sites), thread tapping, and preparation–insertion workflows influence implant placement outcomes. The primary hypotheses were that (1) rCAIS yields similar placement accuracy across site types, but the drills experience higher lateral force at immediate site due to anatomical variations between immediate and healed sites, and (2) thread tapping and fully guided rCAIS will reduce implant positional deviations.

## 2. Materials and Methods

### 2.1. Ethical Approval

The protocol was approved by the Ethics Committee of the School & Hospital of Stomatology, Wuhan University [WDKQ2024] (D01), approval date: 3 January 2024.

### 2.2. Acquisition of Study Model

**Figure 1:**
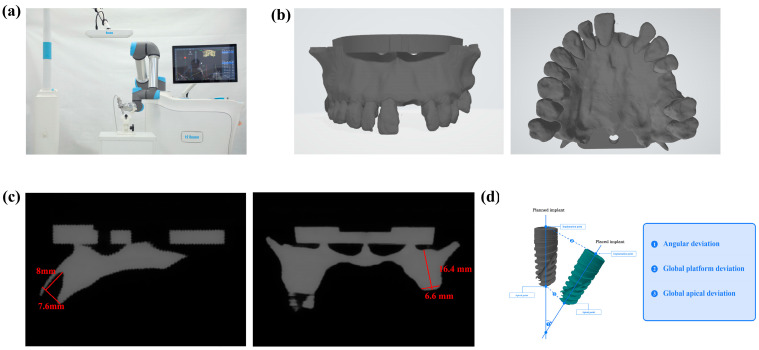
Illustration of the experiment modeling and setting. (**a**) Experimental scene. (**b**) 3D model reconstructed by cone beam CT (CBCT). (**c**) Cone beam CT (CBCT) cross-section of implant sites. (**d**) Scheme of accuracy measurement.

**Figure 2:**
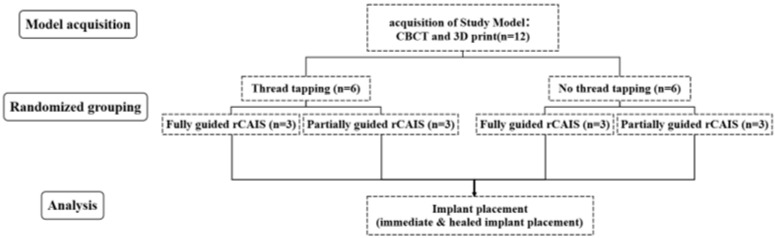
Flow chart of the study.

**Figure 3:**
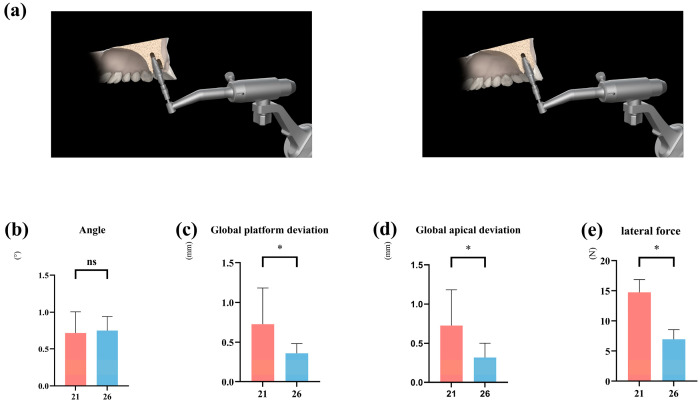
Influence of different implant socket morphologies on robotic implantation accuracy and lateral forces. (**a**) Immediate implant placement (site #21) and healed extraction socket placement (site #26). (**b**–**d**) Comparison of accuracy of different forms of implant sockets placed using implant robot. (**e**) Comparison of lateral force of implants placed using implant robot. (* denotes *p* < 0.05, ns denotes *p* > 0.05).

With the patient’s consent, three-dimensional digital images of the jawbone were acquired using cone beam computed tomography (CBCT, NewTom 5G version FP; NewTom, Verona, Italy). In vitro models of the prospective implantation sites were created using 3D reconstruction techniques, and slices of the surgical area were segmented and 3D printed (Figure 1b and Figure 2). All models were fabricated using Somos EvoLVe 128 resin (Stratasys, Minnetonka, MN, USA)and a single 3D printer (UnionTech D600; UnionTech, Shanghai, China) with a Shore D hardness of 83, as reported in our previous research [11]. The resin material (Shore D 83) provides consistent mechanical resistance suitable for controlled benchtop comparisons; however, it does not replicate the anisotropic or viscoelastic properties of cortical and trabecular bone. Immediate implant sites were designated as FDI tooth position 21, while delayed implants (healing period exceeding three months) were designated as FDI tooth position 26. In this model, the residual alveolar bone height at implant site 21 was 22 mm, and the width was 7.9 mm; the corresponding dimensions for implant site 26 were 16.4 mm in height and 6.6 mm in width (Figure 1c). Two types of implant models were constructed at the different sites. Site 21 was the immediate implant model, where the drilling and implantation were performed on sloped bone walls, experiencing both low and high bone densities across the incline. Site 26 was the delayed implant model; both socket preparation and implant insertion were conducted in homogeneous bone (Figure 3a). The computer randomly assigned 12 models into 4 implant preparation workflows, with 3 models per group, with each model having 2 implant sites with different bone morphology (immediate and healed site). The groups are listed in Table 1. Group allocation was performed using computer-generated randomization (random number sequence generated in Excel using the RAND function). The experimental groups were created by randomly assigning the 12 models to group labels before any implant procedures were performed. Allocation was concealed from the operator until the models were mounted for surgery to minimize selection bias. Because each 3D-printed model inherently contained both predefined implant sites (tooth 21 as an immediate site and tooth 26 as a healed site), randomization could only be applied at the model level, whereas allocation of the two site types within each model was fixed by design.

### 2.3. Sample Size

The preparation strategies were identified as the most influential factors, while angular deviations emerged as the primary indicators. In a preliminary experiment, the angular deviations for the two preparation strategies were 0.71 ± 0.11° and 0.98 ± 0.39°, respectively. There was no statistically significant difference between these deviations. An equivalence test, with a significance level (α) of 0.05, a statistical power of 0.9, an equivalence margin (Δ) of −1~1°, and equal sample sizes in the groups, determined that a sample size of 3 per group was sufficient. This resulted in a total of 12 models.

### 2.4. Implant Site Preparation

The marker (a device utilized in the preoperative calibration of the implant robot) (Remebot, Beijing Ruiyibo Technology Co., Ltd., Beijing, China) was affixed adjacent to the missing tooth using temporary cement (Protemp 4, 3M ESPE). Preoperative CBCT images of the model were then captured with the marker in place. These images were converted to the Digital Imaging and Communications in Medicine (DICOM) format using NNT Viewer software (v.4.20, Quantitative Radiology, Verona, Italy) and imported into the robot planning software, RemebotDent (V 47-2, Remebot, Beijing Ruiyibo Technology Co., Ltd., Beijing, China), for preoperative planning. For the implant site design, the anterior teeth were designed with reference to the choice of implant position in the aesthetic zone and were centrally located in the distal–medial direction, with the platform flush with the palatal alveolar bone and with the long axis parallel and close to the palatal bone wall [12]. In the posterior teeth the proximal and distal mesial were centered, buccolingually oriented, and oriented parallel to the long axis of the expected crown.

The model was secured on a stand, and the implant handpiece was connected to the gripper at the end of the dental robot’s robotic arm (Remebot, Beijing Ruiyibo Technology Co., Ltd.). Marker files in text format were imported into the RemebotDent software. A calibration plate was attached to the handpiece and recorded using an optical tracking locator (camera). The robotic arm maneuvered the calibration plate through six noncoplanar positions around the model, strategically covering the oral implant area to ensure precise spatial positioning. Utilizing the calibration plate, marker file, CBCT images, and optical tracking locator, the robot software established the 3D positional relationships among the robotic arm, the planned implant, and the virtual patient, completing the registration process.

The implant preparation was carried out following the manufacturers’ guidance. Briefly, the preparation was completed as shown in Figure 1a, and a round bur was utilized to penetrate the cortical bone. Sequentially, 2.2 mm, 2.8 mm, and 3.5 mm twist drills were used for drilling. After neck shaping, thread tapping was performed in the thread tapping group, or the implant was inserted directly without thread tapping.

For all groups, the implant site preparation was carried out by the robot system. For the fully robotic-assisted implant placement group, the implant placement was also guided by the robot system, while for the partially robotic-assisted implant placement group, the implant placement was completed freehand by the same surgeon with over 5 years of implant surgery experience. Operator blinding was not feasible because the operator needed to execute the assigned preparation and insertion protocols for each experimental group.

### 2.5. Postoperative Scanning and Analysis

Postoperative scanning and analysis were carried out according to our group’s previous research [11]. After the implant placement was completed, the model was removed from its stand. Subsequent postoperative CBCT scans were then conducted, and the resulting images were converted to DICOM format utilizing NNT Viewer software. These postoperative images were uploaded to RemebotDent, a robotic planning software, where they were blended and compared with preoperative planning images of the implants. This comparison enabled the software to determine several metrics: the angular deviation of the implants, the global deviation at the platform point, and the global deviation at the apical point (Figure 1d). The lateral forces were recorded by the software RemebotDent through force sensors. Data analysis was performed by an independent researcher who was blinded to group allocation. However, complete blinding was not possible: The distinction between immediate and healed sites was visually apparent on the models and thus could not be masked.

All CBCT scans were consistently performed using the same machine (NewTom 5G version FP; NewTom) and under identical settings: 110 kV, 2.3 mA, a voxel size of 0.3 mm, a scan duration of 3.6 s, and a field of view measuring 15 × 12 cm.

### 2.6. Statistical Methods

All statistical analyses were performed in R (version 4.5.1). The outcomes included angular deviation (°), global platform deviation (mm), global apical deviation (mm), and lateral force (N). To account for the paired design in which each model received both immediate (21) and healed (26) implants, linear mixed-effects models (LMMs) were fitted for each outcome, with site (immediate vs. healed), protocol group (four combinations of tapping × guidance), and their interaction as fixed effects, and model ID as a random intercept. Mean ± SD values refer to observed sample distributions (*n* = 3 per condition) in Table 2.

Estimated marginal means (EMMs) were obtained using the emmeans package.

Three levels of inference were performed:

Primary fixed effect: site effect (immediate vs healed)

- Extracted directly from the LMM fixed-effect coefficient.
- Corresponding Cohen’s d values were computed using pooled SD.
- Summary results are reported in Table 3.

Within fully guided rCAIS (no tapping vs. tapping): tapping effect

- Custom contrasts (tapping vs. non-tapping) were applied to EMMs within the fully guided conditions at each site.
- Results are summarized in Table 4.

Within non-tapping protocol: fully guided rCAIS vs. partially guided rCAIS (workflow effect)

- For comparisons of guidance modality, EMM contrasts were computed between fully guided and partially guided conditions within each site.
- Results are presented in Table 5.

All tests were two-sided, and statistical significance was defined as *p* < 0.05.

## 3. Results

### 3.1. Brief Summary of the Experiment

In this study, a total of 12 models were used, with 24 implant sites in 8 groups and 3 implants each per group, as shown in Table 1 and Table 2.

### 3.2. Impact of Implant Socket Morphology on Implant Positional Accuracy and Lateral Forces

Across all protocols combined, healed sites demonstrated substantially lower platform and apical deviations compared with immediate sites (Table 3). Global platform deviation: estimate −0.97 mm, *p* < 0.001, Cohen’s d = 1.10 (large); global apical deviation: estimate −0.95 mm, *p* < 0.001, Cohen’s d = 1.18 (large); angular deviation: no statistically significant difference (+0.22°, *p* = 0.058) (Figure 3b–d).

Regarding lateral forces, measurements from the robotic force feedback indicated that the peak lateral forces encountered during immediate implantation were significantly higher than those at delayed implant sites (Figure 3e). This suggests that immediate implant placement involves greater pushing/lateral forces, leading to transverse displacement of the implant.

### 3.3. Impact of Preparation Strategies on Implant Positional Accuracy and Lateral Forces

**Figure 4:**
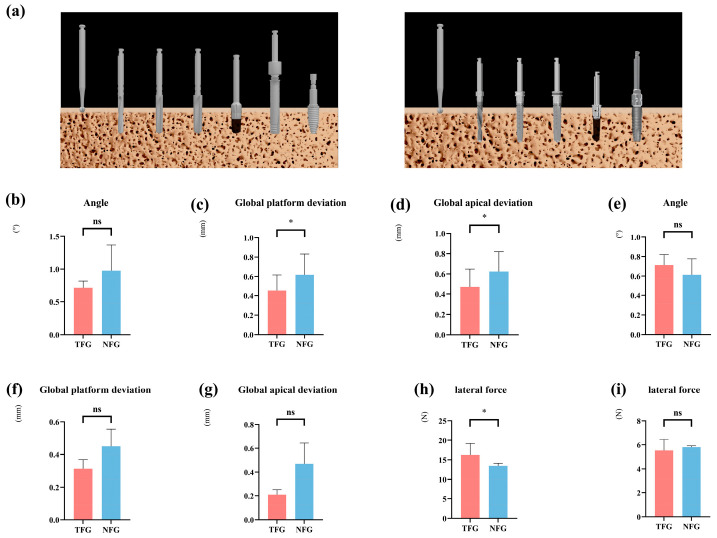
Influence of different preparation strategies on implant accuracy and lateral forces. (**a**) Schematic illustration of implantation with different preparation strategies. (**b**–**d**) Comparison of the accuracy of implantation with and without thread tapping using implant robot-assisted surgery at immediate sites. (**e**–**g**) Comparison of the accuracy of implantation with and without thread tapping using the implant robot at healed sites. (**h**) Comparison of the lateral forces at the immediate implant site using implant robot-assisted surgery for implantation of the implant with or without thread tapping. (**i**) Comparison of the lateral forces using implant robot-assisted surgery for implant placement with or without thread tapping. (TFG: fully guided rCAIS with thread tapping, NFG: fully guided rCAIS for implants without thread tapping), (* denotes *p* < 0.05, ns denotes *p* > 0.05).

Due to the substantial global platform and apical deviations, and the lateral forces observed during the drilling and insertion processes of immediate implants, we investigated whether different preparation strategies could enhance implant accuracy and mitigate the effects of lateral forces. We employed two strategies: one involving thread tapping and another without thread tapping (Figure 4a). Our findings indicate that within the fully guided rCAIS, tapping substantially improved accuracy at the immediate site (platform: −0.98 mm, *p* < 0.0001; apex: −0.96 mm, *p* < 0.0001; angular deviation unchanged, *p* = 0.144). However, at the healed site, the improvement was only modest at the implant apex (−0.27 mm, *p* = 0.023) (Figure 4b–g and Table 4).

### 3.4. Workflow Effect (Fully vs. Partially Guided rCAIS) in a No-Thread Tapping Strategy

**Figure 5:**
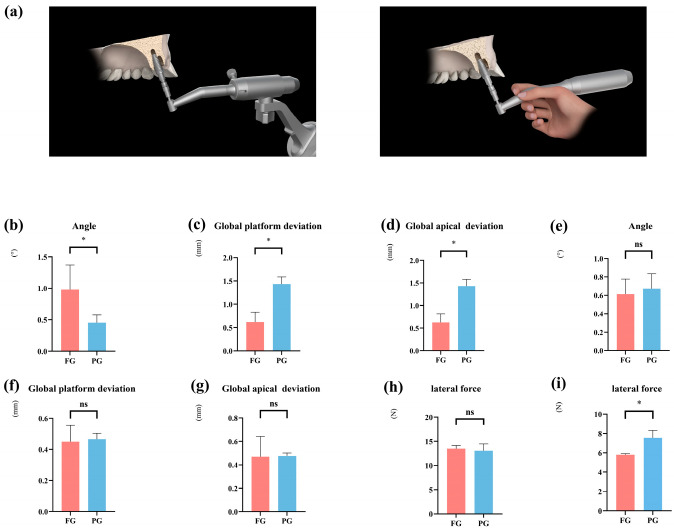
Influence of different guiding modalities on implant accuracy and lateral forces at the immediate and healed implant placement site. (**a**) Schematic illustration of both guided implant placements. (**b**–**d**) Comparison of implant accuracy at the immediate implant site using different guided implant placements with no thread tapping. (**e**–**g**) Comparison of implant accuracy at the healed implant site using different guided implant placement with no thread tapping. (**h**) Comparison of lateral forces in the immediate implant site using implant robot-assisted surgery for implant placement with no thread tapping. (**i**) Comparison of the lateral forces in the healed implant site using implant robot-assisted surgery for implant placement with no thread tapping. (FG: fully guided rCAIS; PG: partially guided rCAIS). (* denotes *p* < 0.05, ns denotes *p* > 0.05).

We subsequently examined the differences in accuracy between robotic and manual implant placement following robotic implant site preparation (Figure 5a). In the context of immediate implants using a no-thread tapping strategy, fully guided rCAIS significantly improved accuracy in immediate sites: global platform deviation: −0.82 mm, *p* < 0.001; global apical deviation: −0.81 mm, *p* < 0.001. However, it produced larger angular deviation (+0.52°, *p* = 0.009). No significant differences were observed in healed sites for accuracy metrics, but the fully guided method significantly reduced insertion force (−1.75 N, *p* = 0.004). This suggests that when employing a no-thread tapping preparation strategy, a full-process rCAIS is still recommended for enhanced accuracy. In terms of lateral forces, no significant differences were observed between the different osteotomy preparation strategies (Figure 5h,i and Table 5).

## 4. Discussion

Utilization of dental robots and robot-assisted dental implant placement marks a huge technological advancement in the field of digital dentistry that improves the accuracy of oral implant placement. Robot-assisted technologies in dentistry have also been used in other fields, including prosthodontics, oral surgery, orthodontics, and endodontics, fostering the development of smart, accurate, and less invasive dental treatments [13]. In dental implantology, robotic systems have shown to bolster clinical outcomes [14]. Nonetheless, further research is still needed to investigate how, for example, bone morphology at the implant site and implant osteotomy preparation procedures affect the accuracy of robotic applications. These studies aim to improve the effectiveness and cost-efficiency of robotic applications in dental implantology.

The robotic arm of the dental robot is equipped with haptics, which can be used to detect forces during the preparation and implant placement processes, allowing the robotic arm to replicate human-like responses to touch and pressure [8,9]. For example, the robot’s force feedback can be used to predict the insertion torque of the implant for future optimization of the robotic surgical procedure with proper primary stability of the implants placed [15].

In this study, we utilized the force feedback feature of the robot to quantify the lateral forces exerted during immediate implant placement and delayed implant placement. This force feedback could be applied in the future to detect bone penetration at the base of the maxillary sinus and be used to simulate the sense of emptiness during the doctor’s manual operations [16]. In the future, robotic force feedback could be used in additional scenarios to help improve surgical accuracy and safety.

Immediate implant placement is gaining popularity due to its shortened overall treatment duration and reduced edentulous period. It is typically recommended to insert implants with a more palatal placement to utilize the remaining bone volume to achieve better initial stability and to avoid the risk of gingival recession associated with implants placed too far buccally [12]. However, during immediate implant placement, achieving optimal accuracy in implant positioning is more challenging compared to delayed implant placement. This is mainly due to the presence of slopes in the bone at the implant site after tooth extraction, which causes the drill and implant to move towards areas of lower bone density and lesser bone volume during the preparation of the implant socket. Consequently, this can lead to a deviation in the implant position from its intended position [3]. Our study found that the lateral forces at immediate implant sites were significantly higher than those at delayed implant sites, a conclusion consistent with prior research demonstrating the tendency of implants placed more facially at the immediate site [3]. Quantitative data on lateral forces in robotic-assisted implant placement appear limited in the existing literature. In this study, we quantified the differences in these lateral forces, providing a cautionary note for clinicians performing freehand placement, especially when performing immediate implant placement. This should prompt further attention to the adjustments needed in the drilling strategy. On the other hand, while the accuracy errors in immediate implantation assisted by robotics were found to be greater than those in delayed implant placement, the error range for robotic implant placement was only 0.59–1.37° (angle), 0.35–0.77 mm (global platform deviation), and 0.33–0.79 mm (global apical deviation), aligning well with prior studies demonstrating that robotic guidance has high accuracy [17]. Some reports have indicated that the robot system represents a higher standard than that of static guides and dynamic navigation [17,18]. However, robots and dynamic navigation have the drawback of requiring daily accuracy and sensitivity for maintenance.

Studies have now shown that compared to traditional drilling protocols, implants placed using tap drilling exhibited higher insertion torque (IT) and implant stability quotient (ISQ) values in bone of varying densities. Researchers found that tap drilling resulted in greater stability indices across all density levels, indicating that tap drilling techniques may enhance implant primary stability [19]. Another study indicated that under-preparation of the implant site diameter can also increase ISQ values and implant insertion torques [20]. Our study revealed that when a tapping strategy is used for implant site preparation, the accuracy and lateral forces do not differ between fully guided rCAIS and partially guided rCAIS. However, when partially guided rCAIS is employed without tapping, the accuracy of implant placement decreases. This further emphasizes the importance of full robotic guidance for enhancing the accuracy and optimization of implant procedures. These findings provide mechanistic evidence to guide future in vivo and clinical trials. The observed accuracy loss and increased lateral forces in immediate sites indicate that clinical protocols may require enhanced osteotomy preparation or force-compensation strategies when using robotic systems.

This study has several limitations. First, its in vitro design limits external validity. Although 3D-printed resin models allow for controlled comparisons, they do not replicate the anisotropic, heterogeneous, and viscoelastic characteristics of human bone, nor can they simulate the effects brought about by the soft tissues in the mouth and saliva; therefore, the results reflect mechanistic behavior under standardized conditions rather than true clinical drilling or insertion resistance. Second, the sample size per condition was small (*n* = 3 implants), and although linear mixed-effects models were used to account for clustering at the model level, model-level random effects were sometimes unstable, and protocol-related comparisons (tapping vs. non-tapping, fully vs. partially guided) should be interpreted as exploratory. Third, accuracy measurements were limited by the 0.3 mm CBCT voxel size and registration workflow. No repeat scans, repeated registrations, or inter-operator reliability assessments were performed, meaning measurement uncertainty may approach the magnitude of the small deviations reported. Additionally, the robotic system was not cross-validated against an external standard, and some variability may exist across units or software versions. Finally, each printed model contributed both an immediate and a healed site, and unmeasured within-model correlation may persist despite mixed-effects adjustment. The negative or non-significant findings observed may represent true effects or be influenced by the limited statistical power. Larger studies—including bone analogs with realistic mechanical properties, in vivo experiments, and clinical trials—are required to confirm the accuracy and force behaviors observed here.

## 5. Conclusions

Within the limits of this benchtop model, immediate implant placement showed lower accuracy than healed-site placement, although both remained within a generally high-accuracy range. In addition, fully guided rCAIS appeared to improve accuracy in immediate sites without thread tapping. These findings should be interpreted cautiously given the small sample size and in vitro setting.

## Figures and Tables

**Table 1:** Information about groups.

Group	Procedure	Implants
1	Immediate site + Thread tapping + Fully guided rCAIS	3
2	Immediate site + No thread tapping + Fully guided rCAIS	3
3	Immediate site + Thread tapping + Partially guided rCAIS	3
4	Immediate site + No thread tapping + Partially guided rCAIS	3
5	Healed site + Thread tapping + Fully guided rCAIS	3
6	Healed site + No thread tapping + Fully guided rCAIS	3
7	Healed site + Thread tapping + Partially guided rCAIS	3
8	Healed site + No thread tapping + Partially guided rCAIS	3

**Table 2:** Implant surgery deviations and lateral force for groups.

Groups	1	2	3	4	5	6	7	8
Angle (°)	0.71 ± 0.11	0.98 ± 0.39	0.72 ± 0.28	0.45 ± 0.12	0.71 ± 0.11	0.61 ± 0.16	0.75 ± 0.35	0.67 ± 0.16
Global platform deviation (mm)	0.45 ± 0.16	0.62 ± 0.22	0.40 ± 0.05	1.43 ± 0.15	0.31 ± 0.06	0.45 ± 0.11	0.21 ± 0.02	0.47 ± 0.04
Global apical deviation (mm)	0.47 ± 0.18	0.62 ± 0.19	0.38 ± 0.11	1.43 ± 0.15	0.21 ± 0.04	0.47 ± 0.17	0.15 ± 0.04	0.48 ± 0.03
Lateral force (N)	16.26 ± 2.91	13.47 ± 0.64	16.17 ± 1.08	13.04 ± 1.42	5.53 ± 0.91	5.80 ± 0.11	9.06 ± 0.14	7.55 ± 0.78

**Table 3:** Mixed-effects model results for site effect (immediate vs. healed).

Outcome	Estimate (Healed–Immediate)	SE	t Value	*p*-Value	Effect Size (d)	Interpretation
Global platform deviation (mm)	−0.97	0.098	−9.91	<0.001	1.10 (large)	Healed is more accurate.
Global apical deviation (mm)	−0.95	0.106	−8.96	<0.001	1.18 (large)	Healed is more accurate.
Angular deviation (°)	0.22	0.099	2.21	0.058	−0.13 (negligible)	No meaningful difference
Lateral force (N)	−5.49	0.94	−5.86	0.0004	4.11 (extremely large)	Healed requires far less force.

**Table 4:** Fully guided condition: tapping vs. no tapping.

Outcome	Site	Tapping (Mean ± SD)	Non-Tapping (Mean ± SD)	Estimate (T − NT)	*p*-Value	Interpretation
Global platform deviation (mm)	Immediate	0.45 ± 0.16	0.62 ± 0.22	−0.98	<0.0001	Tapping greatly improves accuracy.
	Healed	0.31 ± 0.06	0.45 ± 0.11	−0.15	0.136	Not significant
Global apical deviation (mm)	Immediate	0.47 ± 0.18	0.62 ± 0.19	−0.96	<0.0001	Tapping improves depth accuracy.
	Healed	0.21 ± 0.04	0.47 ± 0.17	−0.27	0.023	Small but significant
Angle deviation (°)	Immediate	0.71 ± 0.11	0.98 ± 0.39	+0.26°	0.144	Not significant
	Healed	0.71 ± 0.11	0.61 ± 0.16	+0.04°	0.813	No difference
Lateral force (N)	Immediate	16.26 ± 2.91	13.47 ± 0.65	+3.22	0.008	Tapping requires more force.
	Healed	5.53 ± 0.91	5.80 ± 0.11	−2.02	0.076	Not significant

**Table 5:** Comparison of fully vs partially guided rCAIS in immediate and healed sites with no-tapping protocol. Values reported as mean ± SD (*n* = 3 per condition). Effects represent estimated marginal means contrasts from linear mixed-effects models with site, group, and their interaction as fixed effects and model as random intercept. Negative estimates favor the fully guided method for accuracy outcomes (lower deviation = better).

Outcome	Comparison	Mean ± SD	Effect (Estimate)	*p*-Value	Interpretation
Global platform deviation (mm)	Fully vs. partially (immediate)	0.62 ± 0.22 vs. 1.43 ± 0.15	−0.82	<0.001	Fully guided markedly improves accuracy in immediate sites.
	Fully vs. partially (healed)	0.45 ± 0.11 vs. 0.47 ± 0.04	−0.02	0.93	No meaningful difference in healed sites
Global apical deviation (mm)	Fully vs. partially (immediate)	0.62 ± 0.19 vs. 1.43 ± 0.15	−0.81	<0.001	Fully guided greatly improves apical accuracy in immediate sites.
	Fully vs. partially (healed)	0.47 ± 0.17 vs. 0.48 ± 0.03	−0.01	0.98	No difference
Angular deviation (°)	Fully vs. partially (immediate)	0.98 ± 0.39 vs. 0.45 ± 0.12	+0.52°	0.009	Fully guided increases angular deviation (worse).
	Fully vs. partially (healed)	0.61 ± 0.16 vs. 0.67 ± 0.16	−0.06°	0.71	No difference
Lateral force (N)	Fully vs. partially (immediate)	13.47 ± 0.64 vs. 13.04 ± 1.42	+0.43	0.69	No measurable difference
	Fully vs. partially (healed)	5.80 ± 0.11 vs. 7.55 ± 0.78	−1.75	0.004	Fully guided significantly reduces insertion force.

## Data Availability

The original contributions presented in this study are included in the article. Further inquiries can be directed to the corresponding authors.

## Abbreviations

The following abbreviations are used in this manuscript:

CAIS	Computer-aided implant surgery
NFG	Fully guided rCAIS without thread tapping
TFG	Fully guided rCAIS with thread tapping
FG	Fully guided rCAIS
PG	Partially guided rCAIS
